# Inflammatory markers assessment in an animal model of intracranial hypertension: a randomized trial

**DOI:** 10.1186/s40635-021-00408-5

**Published:** 2021-08-23

**Authors:** Marcelo Prudente do Espírito Santo, Caroline Silvério Faria, Davi Jorge Fontoura Solla, Leonardo Zumerkorn Pipek, Alessandro Rodrigo Belon, Brasil Ping Jeng, Almir Ferreira de Andrade, Manoel Jacobsen Teixeira, Wellingson Silva Paiva

**Affiliations:** Division of Neurosurgery, University of São Paulo Medical School, São Paulo, Brazil

**Keywords:** Inflammatory markers, Intracranial hypertension, Animal model, Cytokines, Inflammation

## Abstract

**Background:**

Intracranial hypertension (ICH) is a common final pathway of most neurosurgical pathologies and leads to poor prognosis if not detected and treated properly. Inflammatory markers have been assessed in clinical scenarios of neurological injuries, in which systemic and brain tissue aggressions may introduce bias. There is a lack of studies under controlled settings to isolate the ICH effect on inflammation. This study aims to evaluate the effects of ICH on the serum concentration of cytokines as biomarkers of neuroinflammation in an experimental model which isolates ICH from potential confounding variables.

**Methods:**

An established model of ICH using an intracerebral pediatric bladder catheter and a multisensor intraparenchymal catheter was used in adult pigs (*Sus domesticus*). The animals were randomly allocated to 2 groups based on the catheter balloon volume used to simulate the ICP increase (4 ml or 7 ml). Cytokines were measured in 4 timepoints during the experiment: (1) 15 min before balloon insufflation; (2) 5 min after insufflation; (3) 125 min after insufflation; (4) 60 min after deflation. The following cytokines were measured IL-1α; IL-1β; IL–1ra; IL-2; IL-4; IL-6; IL-8; IL-10; IL-12; IL-18; TNFα. Generalized estimating equations were modeled to compare the ICP and cytokines values between the groups along the experiment. The study sample size was powered to detect interactions between the groups and the study moments with an effect size (f) of at least 0.3. The ARRIVE checklist was followed.

**Results:**

A total of 20 animals were studied (10 in each group, 4 ml or 7 ml balloon volume insufflation). The animal model was successful in increasing the ICP along the moments of the experiment (*p* < 0,001) and in creating an ICP gradient between the groups (*p* = 0,004). The interaction term (moment × group) was also significant (*p* < 0,001). There was a significant association between ICP elevation and most cytokines variation. The cytokines IL-1α, IL-1β, IL1-ra, IL-6, IL-12, and IL-18 increased, whereas IL-2, IL-4, and TNF-α decreased. IL-10 did not vary significantly in response to the ICP elevation.

**Conclusion:**

The serum concentration of cytokines varied in response to intracranial hypertension. The study demonstrated the specific changes in each cytokine after intracranial hypertension and provides key information to guide neuroinflammation clinical research. The proposed experiment was successful as an animal model to the study of neuroinflammation biomarkers

**Supplementary Information:**

The online version contains supplementary material available at 10.1186/s40635-021-00408-5.

## Introduction

Intracranial hypertension (ICH) is the common final pathway of most neurosurgical pathologies. Disturbances of the cerebrospinal fluid (CSF) circulation, diffuse and focal traumatic injuries, and intracranial expansive lesions tend to increase intracranial pressure (ICP) [1]. ICH leads to a sequence of events which affect brain tissue such as ischemia, edema, and necrosis. These events release biomarkers which can be measured in samples of brain tissue, CSF, and even peripheral blood due to concomitant disruption of the blood–brain barrier (BBB). Biomarker dosage can be useful as an indicator of biological processes, whether natural, pathological, or in response to a therapeutic intervention [2]. One of the main advantages is that they can be readily detected with potentially high sensitivity and specificity [3]. Understanding the dynamics of biomarkers promises to aid in the diagnosis, management, and prognosis of brain injuries.

Experimental models of traumatic brain injury (TBI) have demonstrated several inflammatory mediators expressed in brain tissue, some of them with concentration peaks at the order of 10^3^ beyond the usual levels [4]. The magnitude and dynamics of these mediators’ expression may reveal information about the primary lesion and the sequence of events that follow. Among these biomarkers, cytokines have been studied as markers of brain injury. These are small proteins with short half-lives produced by leukocytes and glial cells and quickly released in response to tissue aggression. A large number of cytokines, many with overlapping functions, form a complex network of inflammatory mediators. Cytokines that initiate or propagate an inflammatory response are called proinflammatory, while those inhibiting it are called anti-inflammatory [5]. The expression profile of each cytokine can be easily and quickly measured through immunological assays.

Inflammatory markers have already been studied in TBI and subarachnoid hemorrhage (SAH). In these scenarios, the cytokines variation is confounded by the inflammatory effect of systemic and brain tissue aggressions [6]. Thus, there is a lack of studies under controlled settings evaluating the ICH effect on the levels of biomarkers. Animal experimental models are better suited for such purpose than clinical studies. However, published ICH experimental models usually describe anatomically irreversible brain injury methods, which make it difficult to study systemic parameters, ICP, and biomarkers after an intervention to reduce ICP [7, 8].

Our objective is to evaluate the effect of intracranial hypertension on the serum concentration of cytokines as biomarkers of neuroinflammation in an experimental model.

## Methods

### Setting

All animal experiments were performed in the animal laboratories at the University of São Paulo, Brazil. The animal model was adapted from an established model of intracerebral expansive process using a pediatric bladder catheter and a multisensor intraparenchymal catheter (Neurovent-PTO, Raumedic^®^, Munchberg, Germany) [9].

### Ethical considerations

All experimental protocols were approved by the Animal Ethics Committee (FMUSP, Brazil). All procedures were guided by the National Council for the Control of Animal Experimentation (CONCEA). We emphasize that in no stage of the experimentation the animals were subjected to conditions of suffering or pain. Principles of the 3Rs were implemented, and humane endpoints were applied. The experiments adhered to the Animal Research: Reporting of In Vivo Experiments guidelines [10].

### Research animals

Male (*n* = 10) and female (*n* = 10) adult pigs (*Sus domesticus*), hybrids of the Landrace, Duroc, and Pietrain breeds were used for the experiment. The selected animals were around 60 days old and had between 17.0 and 42.3 kg of body weight.

### Study design

**Fig. 1:**
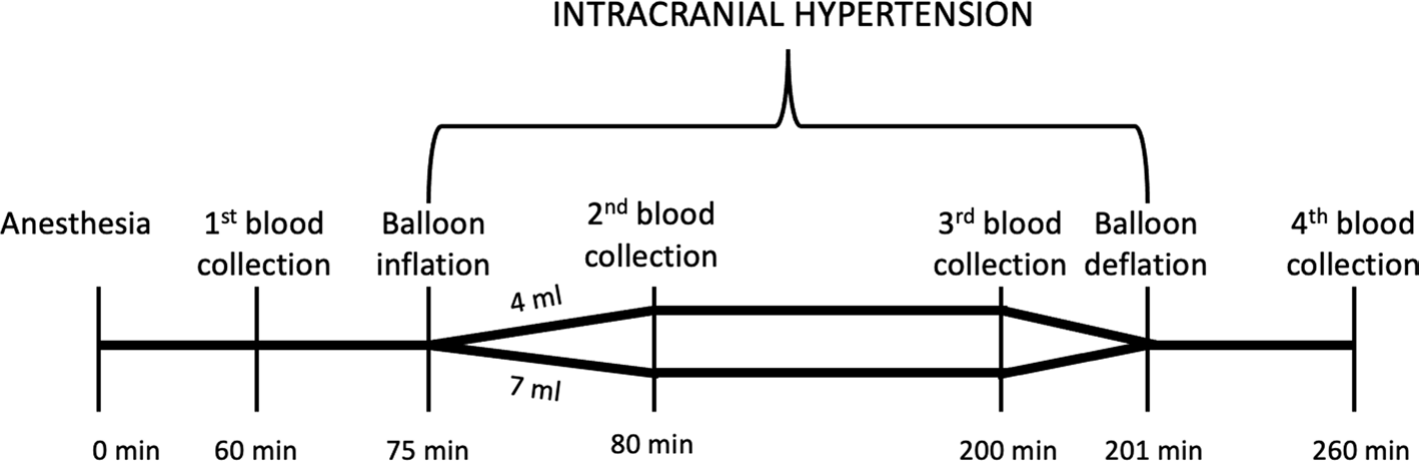
Study design. Blood for cytokines analysis was collected before (1st), during (2nd/3rd) and after (4th) simulating intracranial hypertension

The animals (*n* = 20) were randomly allocated using a computer random group generator to one of 2 groups based on the volume used to simulate the increase in the intracranial pressure: 4 ml or 7 ml. All authors were aware of the group allocation at the different stages of the experiment. Cytokines were collected before, during, and after the simulated intracranial hypertension. Figure 1 displays the study design for the experiment.

### Sample size

The study sample size had 80% power to detect interactions between the two groups and the study four moments with an effect size (*f*) of at least 0.3, assuming alpha error probability 0.05 and correlation among repeated measures 0.5.

### Animal preparation, anesthesia, and euthanasia

The pigs were fasted for 12 h, with free access to water up to one hour before the experiment.

Intramuscular ketamine (Ketamin-S^®^, Cristália—5 mg/kg) and midazolam (Dormire^®^, Cristália—0.25 mg/kg) were used as a pre-anesthetic medication. Venous access was established through catheterization of the marginal vein of the ear using a 20 or 22 caliber vascular catheter (BD Insyte^®^, Becton Dickinson).

Once venous access was established through catheterization of the marginal vein of the ear, anesthetic induction was performed with the application of propofol (Provive^®^ 1%, Claris—5 mg/kg), associated with an initial volume of 20 mL/kg of physiological solution (NaCl 0.9%) for compensation for volume loss related to fasting.

Animals were intubated with a 6-mm-diameter endotracheal tube (Portex^®^). IV propofol (3 mg/kg/h) and Fentanyl (Fentanest^®^, Cristália—bolus of 5 µg/kg + 0.4 µg/kg/min) were used for maintaining anesthesia and analgesia.

The animals were mechanically ventilated, cycled to volume (Ventilador Dixtal^®^ 5010). Ventilation adequacy was assessed by continuous measurement of the final pressure of expired carbon dioxide (EtCO_2_), peripheral hemoglobin saturation by pulse oximetry (SpO_2_), and measurement of gases in arterial blood samples.

The right femoral artery was catheterized to monitor mean arterial pressure (MAP), systolic (SBP), and diastolic (DBP). Hemodynamic data were collected using a multiparameter monitor (Monitor Portal DX 2020, Dixtal^®^). From this venous access located in the right femoral artery, arterial blood gas tests (0.3 ml volume samples) were collected to monitor ventilatory and physiological parameters, totaling four collections of arterial blood gases during the procedure.

Throughout the experiment, the animals were kept hydrated with saline solution (NaCl 0.9%) at a rate of 5 ml/kg/h. Bladder catheterization by cystostomy was performed to monitor diuresis and optimize fluid balance.

The central temperature was maintained between 37 and 38 ºC, with the use of a thermal mattress and previously heated hydration solutions.

The animals were killed with propofol (20 mg/kg) and fentanyl (10 mg/kg), followed by the intravenous administration of 20 ml of 19.1% potassium chloride solution.

### Intracranial hypertension simulation and cytokines analysis

The increase in intracranial pressure was achieved by insufflating a pediatric bladder catheter to simulate an intracerebral expansive lesion. A multisensor intraparenchymal catheter was used to monitor intracerebral parameters.

The procedure started with a frontotemporal skin incision to expose the animal’s right hemi-cranium. After rotation of the skin flap, bone trepanation was performed, located 1 cm lateral to the sagittal suture and 1 cm anterior to the coronal suture, through which the intraparenchymal catheter with a sensor for ICP, tissue oxygen pressure (PtiO_2_p) and brain temperature was introduced (Neurovent-PTO, Raumedic^®^, Munchberg, Germany). The tip of this catheter was positioned in the white matter of the right frontal lobe at a depth of approximately 10 mm in relation to the edge of the trepanation.

A second bone trepanation allowed the introduction of a pediatric bladder catheter sized 8 Fr. The incision was 1 cm lateral to the sagittal suture and 1 cm posterior to the coronary suture, with a diameter of 3 mm, tilted 20 degrees in relation to the lateral plane, reaching a depth of 2 cm. The tip of the catheter was positioned next to the right frontal white matter.

ICP increase was achieved by insufflating the bladder catheter with saline. The filling was controlled with the use of an automatic mechanical infusion pump (B. Braun Melsungen AG, Melsungen, Germany), programmed to reach the desired volume for each group, 4 ml or 7 ml, in 15 min.

Other parameters important for hemodynamic control were also registered, including body temperature, heart rate (HR), blood pressure, oxygen saturation (SO2), and expired carbon dioxide (EtCO_2_).

Peripheral blood samples were collected for arterial blood gases and cytokine analysis. A part of the sample was separated in a tube containing EDTA anticoagulant and stored in a freezer at – 80 ºC for the dosage of cytokines. The impact of intracranial hypertension was assessed in 4 moments: (1) 15 min before insufflation; (2) 5 min after insufflation; (3) 125 min after insufflation; (4) 60 min after deflation.

Plasma samples were prepared for analysis in a 96-well plate, using a customized Milliplex MAP^®^ Porcine Cytokine/Chemokine Magnetic Bead^®^ swine cytokine panel (Millipore Corp., Billerica, USA). The following biomarkers were then quantified using a Magpix^®^ analytical test instrument (Luminex Corp., Austin, USA): IL-1α; IL-1β; IL–1ra; IL-2; IL-4; IL-6; IL-8; IL-10; IL-12; IL-18; TNFα.

### Statistical analysis

For descriptive purposes, categorical variables were presented through relative and absolute frequencies and compared using the Chi-squared or Fisher exact test, as appropriate. Continuous variable distributions were assessed for normality by skewness and kurtosis and by graphical methods. Those with normal distribution were presented as mean and standard deviation and compared by the independent samples Student *T*. Otherwise, they were presented as medians and quartiles and compared by the Mann–Whitney nonparametric test. There were no missing data for the baseline physiologic variables or any of the cytokines or ICP at any moment, except for one animal from the 7-ml group that died before the final cytokine dosage.

Generalized estimating equations (GEE) were modeled to compare ICP and cytokine values between groups along the experiment. For the GEE, it was assumed a normal marginal distribution for the ICP and a gamma distribution for the cytokines (owing to the non-normal distribution of most of them), an identity link function for both ICP and cytokines, and an auto-regressive first-order correlation matrix between the experiment moments. An interaction term between group and moment was introduced as a proxy to assess a possible “dose-dependent response” between ICP and cytokine variation, assuming higher ICP values on the 7-ml group compared to the 4-ml group. Also, the association between ICP and cytokines variation was directly evaluated with a different set of GEEs modeled with each cytokine as a dependent variable and the ICP as an independent variable. To assess and compare the effect sizes of the cytokines variation, their values were logarithmically transformed and standardized as *z* scores regarding the baseline as the reference. Thus, although each cytokine may have a different scale, we could compare them by a standardized parameter.

All tests were 2-sided and final *p* values under 0.05 were considered statistically significant. All analyses were conducted with the SPSS software (IBM Corp. SPSS Statistics para Windows, version 24.0. Armonk, NY).

## Results

### Baseline characteristics

Among the 20 studied animals (mean weight 22,9 ± 8,1 kg, 10 females), no significant baseline differences were found between the groups, except for a tendency for higher male proportion (70,0 vs 30,0%, *p* = 0,074) and smaller DBP (61,2 ± 12,8 vs. 72,6 ± 11,4, *p* = 0,049) for the 4-ml group (). All other parameters (SBP, MAP, end-tidal CO_2_, temperature, heart rate, SatO_2_, brain temperature and tissue oxygen pressure) were similar. Detailed information is presented in Additional file 1.

### Intracranial hypertension simulation

**Fig. 2:**
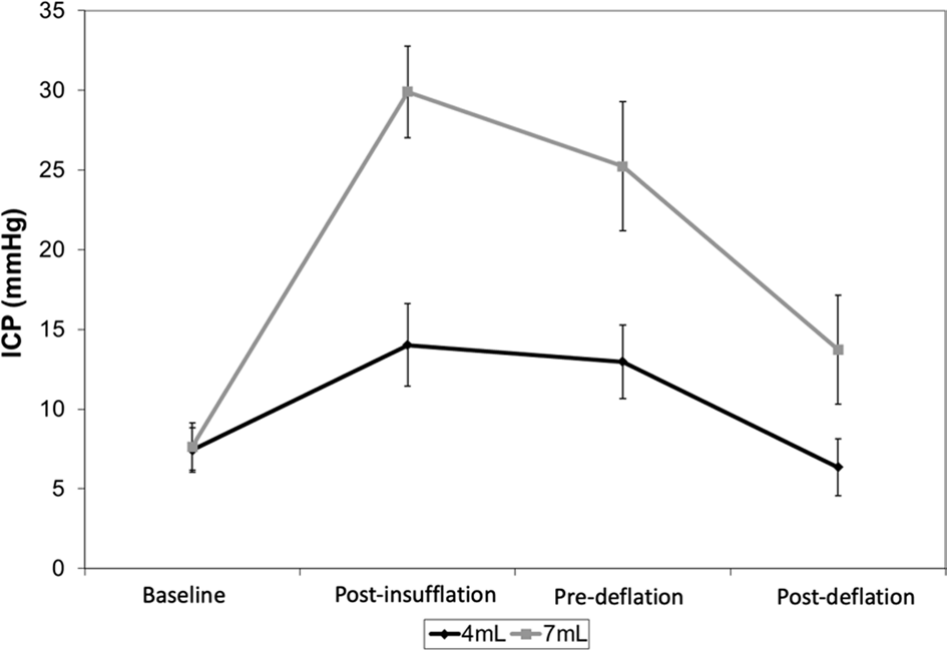
Intracranial pressure for both groups along the experiment moments (mean and standard error)

The ICP increased after the balloon insufflation, underwent relative stabilization until the pre-deflation moment, and then fell after the deflation. The ICP mean (± standard deviation) at baseline, post-balloon insufflation, pre-deflation, and post-deflation moments for the 4-ml group were, respectively, 7.4 ± 4.4, 14.0 ± 8.2, 13.0 ± 7.3 and 6.4 ± 5.6, and, for the 7-ml group, 7.7 ± 4.7, 29.9 ± 9.1, 25.2 ± 12.8 and 13.7 ± 10.3 (Fig. 2). Thus, the animal model was successful in varying the ICP along the moments (*p* < 0,001) and in creating an ICP gradient between the groups (*p* = 0,004). The interaction term (moment × group) was also significant (*p* < 0,001).

### Biomarkers

Considering the hypothesis that, once triggered by the ICP elevation, the inflammatory response would continue for at least a period even after the ICP was lowered (by balloon deflation), we excluded the post-deflation moment to analyze the association between ICP and cytokines values.

**Fig. 3:**
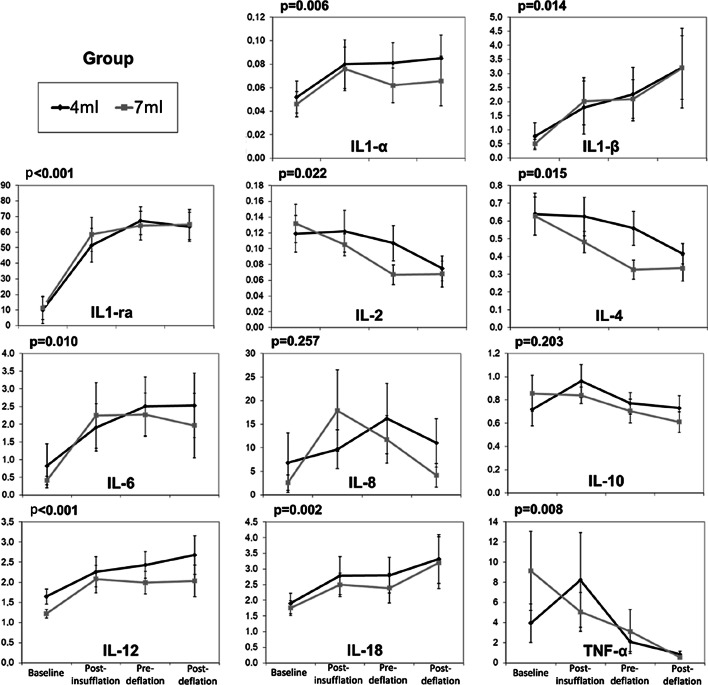
Cytokine values for both groups along the experiment moments (mean and standard error). *p* values refer to the variation along the moments

Figure 3 presents the cytokine values for both groups along the experiment moments, which varied significantly, except for IL-8 and IL-10. The cytokines IL-1α, IL-1β, IL1-ra, IL-6, IL-12, and IL-18 increased, whereas IL-2, IL-4, and TNF-α decreased. There was no statistical difference between the groups, nor interaction between the moment (baseline, pre-insufflation and pre-deflation for both groups combined) and the group.

Detailed information for each cytokine is presented in Table 1.

**Table 1:** Cytokines values along the experiment according to group and moment (*n* = 10 per group)

Group	Cytokine	Moment			*p* values
		Baseline	Pre-insufflation	Pre-deflation	
IL-1α					
4 ml	Median (quartiles)	0,04 (0,03–0,07)	0,05 (0,03–0,13)	0,07 (0,04–0,12)	Group, *p* = 0,617 Moment, *p* = 0,006 Interaction, *p* = 0,848
	Min; max	0,01; 0,14	0,03; 0,20	0,02; 0,17	
7 ml	Median (quartiles)	0,04 (0,02–0,06)	0,05 (0,05–0,06)	0,05 (0,03–0,06)	
	Min; max	0,01; 0,13	0,03; 0,19	0,02; 0,16	
IL-1β					
4 ml	Median (quartiles)	0,27 (0,20–0,44)	0,65 (0,37—1,18)	1,44 (0,51–2,49)	Group, *p* = 0,934 **Moment, *p* = 0,014** Interaction, *p* = 0,889
	Min; max	0,11; 5,05	0,12; 9,74	0,11; 10,1	
7 ml	Median (quartiles)	0,40 (0,20–0,55)	0,97 (0,39–1,39)	1,32 (0,59–2,80)	
	Min; max	0,13; 1,85	0,31; 8,3	0,13; 6,06	
IL-1ra					
4 ml	Median (quartiles)	0,58 (0,37–1,07)	56,1 (24,7–73,3)	72,1 (54,9–84,7)	Group, *p* = 0,919 **Moment, *p* < 0,001** Interaction, *p* = 0,996
	Min; max	0,3; 86,4	0,4; 101,4	2,1; 101,8	
7 ml	Median (quartiles)	0,87 (0,31–2,52)	64,1 (51,7–76,7)	70,8 (49,4–87,6)	
	Min; max	0,2; 70,6	0,5; 109,4	0,4; 94,9	
IL-2					
4 ml	Median (quartiles)	0,12 (0,06–0,17)	0,11 (0,07–0,16)	0,09 (0,05–0,16)	Group, *p* = 0,543 **Moment, *p* = 0,022** Interaction, *p* = 0,327
	Min; max	0,02; 0,27	0,02; 0,30	0,02; 0,20	
7 ml	Median (quartiles)	0,13 (0,07–0,19)	0,11 (0,06–0,14)	0,05 (0,04–0,08)	
	Min; max	0,03; 0,25	0,04; 0,17	0,03; 0,16	
IL-4					
4 ml	Median (quartiles)	0,59 (0,32–0,89)	0,52 (0,44–0,82)	0,51 (0,32–0,85)	Group, *p* = 0,166 **Moment, *p* = 0,015** Interaction, *p* = 0,397
	Min; max	0,16; 1,39	0,16; 1,29	0,16; 1,04	
7 ml	Median (quartiles)	0,60 (0,36–0,78)	0,49 (0,35–0,68)	0,25 (0,23–0,41)	
	Min; max	0,18; 1,20	0,18; 0,74	0,19; 0,74	
IL-6					
4 ml	Median (quartiles)	0,14 (0,11–0,43)	1,21 (0,20–3,12)	1,70 (0,22–5,20)	Group, *p* = 0,837 **Moment, *p* = 0,010** Interaction, *p* = 0,922
	Min; max	0,03; 6,39	0,05; 6,04	0,04; 7,07	
7 ml	Median (quartiles)	0,22 (0,11–0,89)	1,62 (0,49–2,18)	2,27 (0,37–3,24)	
	Min; max	0,06; 1,03	0,12; 10,11	0,04; 5,86	
IL-8					
4 ml	Median (quartiles)	0,17 (0,08–0,30)	3,54 (0,22–14,8)	1,38 (0,20–39,57)	Group, *p* = 0,766 Moment, *p* = 0,257 Interaction, *p* = 0,679
	Min; max	0,04; 63,7	0,03; 40,3	0,04; 58,2	
7 ml	Median (quartiles)	0,40 (0,16–1,07)	11,4 (0,82–19,5)	3,02 (0,89–21,85)	
	Min; max	0,1; 16	0,2; 92,1	0,1; 42,9	
IL-10					
4 ml	Median (quartiles)	0,62 (0,41–1,02)	0,86 (0,70–1,41)	0,88 (0,65–1,02)	Group, *p* = 0,765 Moment, *p* = 0,203 Interaction, *p* = 0,505
	Min; max	0,18; 1,48	0,3; 1,67	0,24; 1,05	
7 ml	Median (quartiles)	0,68 (0,49–1,06)	0,79 (0,69–1,08)	0,66 (0,46–0,81)	
	Min; max	0,29; 1,85	0,53; 1,23	0,29; 1,34	
IL-12					
4 ml	Median (quartiles)	1,59 (1,18–1,85)	2,42 (1,10–3,19)	2,63 (1,34–3,24)	Group, *p* = 0,281 **Moment, *p* < 0,001** Interaction, *p* = 0,793
	Min; max	0,9; 2,7	1; 4,4	0,9; 3,9	
7 ml	Median (quartiles)	1,27 (0,96–1,49)	2,12 (1,16–2,40)	2,13 (1,40–2,46)	
	Min; max	0,7; 1,7	0,8; 4,4	0,6; 3,3	
IL-18					
4 ml	Median (quartiles)	1,45 (1,23–2,46)	2,08 (1,16–4,06)	2,41 (1,35–4,11)	Group, *p* = 0,732 Moment, *p* = 0,002 Interaction, *p* = 0,913
	Min; max	0,7; 4	0,8; 6	0,6; 6,2	
7 ml	Median (quartiles)	1,73 (1,23–2,44)	2,04 (1,76–3,38)	2,24 (1,14–2,76)	
	Min; max	0,7; 3	3; 5,1	0,6; 5,5	
TNF-a					
4 ml	Median (quartiles)	0,99 (0,11–5,13)	2,21 (0,22–3,01)	0,67 (0,16–1,87)	Group, *p* = 0,747 Moment, *p* = 0,008 Interaction, *p* = 0,306
	Min; max	0,03; 17	0,02; 42,7	0,02; 7,9	
7 ml	Median (quartiles)	1,95 (0,15–17,6)	2,59 (0,74–7,84)	0,77 (0,46–1,39)	
	Min; max	0,1; 35,8	0,2; 17,1	0,2; 22,7	

As presented in Table 2, there was a significant association between ICP elevation and most cytokines variation. Only IL-2, IL-4, and IL-10 variations did not reach statistical significance in response to ICP elevation. IL-2 and IL-4 analyses might have been underpowered.

**Table 2:** Generalized estimating equations results for the cytokines response to ICP elevation

Cytokine	ICP (each 1 mmHg)		Wald statistics	*p*-value
	Coefficient	Standard error		
IL-1α	0,001	0,001	9,68	0,002
IL-1β	0,082	0,028	8,48	0,004
IL-1ra	2,180	0,650	11,18	0,001
IL-2	− 0,001	0,001	2,47	0,116
IL-4	− 0,005	0,003	3,34	0,068
IL-6	0,082	0,032	6,58	0,010
IL-8	0,690	0,270	6,43	0,011
IL-10	− 0,003	0,004	0,49	0,483
IL-12	0,032	0,010	10,63	0,001
IL-18	0,036	0,014	6,79	0,009
TNF- α	− 0,151	0,040	14,19	< 0,001

*ICP* intracranial pressure. One degree of freedom for each model

The cytokines that presented the greater standardized variation (z-score, baseline reference) were IL-1ra, IL-1β, IL-6, and IL-12 (Table 3).

**Table 3:** Cytokines standardized (*z*-score) mean variation along the experiment (reference: baseline)

Cytokine	Moment		
	Post-insufflation	Pre-deflation	Post-deflation
IL-1α	0,65 ± 0,84	0,54 ± 0,90	0,51 ± 1,02
IL-1β	1,03 ± 1,30	1,26 ± 1,42	1,49 ± 1,64
IL-1ra	1,67 ± 0,89	1,89 ± 0,74	1,89 ± 0,69
IL-2	− 0,12 ± 0,95	− 0,52 ± 0,92	− 0,78 ± 0,89
IL-4	− 0,17 ± 0,88	− 0,59 ± 0,93	− 0,78 ± 0,79
IL-6	1,15 ± 1,13	1,17 ± 1,35	0,97 ± 1,41
IL-8	1,03 ± 1,15	0,85 ± 1,24	0,53 ± 1,15
IL-10	0,37 ± 0,67	0,01 ± 0,78	− 0,15 ± 0,80
IL-12	0,99 ± 1,51	1,08 ± 1,50	1,11 ± 1,82
IL-18	0,66 ± 1,22	0,53 ± 1,43	0,85 ± 1,66
TNF-α	0,13 ± 0,91	− 0,25 ± 0,77	− 0,54 ± 0,58

Data are presented as *z*-score mean ± standard deviation

## Discussion

Intracranial hypertension and cerebral hypoperfusion can be understood as secondary insults after TBI and their prevention or mitigation are critical for the management of these patients [11]. Numerous studies have consistently shown that early recognition and treatment of these complications lead to better outcomes [12]. Our study sought to evaluate the association between ICP elevation and serum inflammation biomarkers variation, such as cytokines.

The use of an experimental animal model makes it possible to create a controlled environment and minimize confounding variables usually present in clinical studies. Factors such as hypotension, hypoxia, and trauma to other organs may trigger inflammatory responses that influence serum cytokines levels and hamper the interpretation of results. Most published experimental models are based on intracerebral injection of blood, which is useful for studying the pathophysiological effects of spontaneous intracerebral hematoma associated with elevated ICP [7]. Some other experimental models try to reproduce different TBI injury subtypes, such as brain contusion [8]. These latter approaches present the disadvantage of initiating an inflammatory process secondary to the presence of blood in the parenchyma or to the trauma and brain tissue aggression itself and finally leading to cytokines release. In addition, these models do not permit the reversal of the process causing ICH, which would prevent the assessment of cytokines variation dynamics along ICP changes.

However, it should be noted that although the balloon inflation mechanism used in our experiment allows a controlled variation of the ICP without the interference of mechanisms causing the inflammatory response, there is brain tissue injury at the end.

The influence of systemic conditions on cytokine is illustrated by Hergenroeder et al. [13]. The study showed changes in IL-6 in patients with TBI and in orthopedic injury in the absence of TBI. This interleukin could predict ICH in patients with TBI, but also increased in those with orthopedic injuries.

Our experiment achieved the ICP variation required for the study of cytokines, both along the moments of the study within each group, and between the groups in which the vesical balloon was inflated by 4 ml and 7 ml. This result validates the model as the basis of our study.

Although there was substantial variability in the cytokines levels throughout the experiment (which was associated with a significant ICP elevation for each 1 mmhHg, as shown in Table 2), there was no significant difference in the cytokines levels between the 2 specific values chosen for each group (4-ml or 7-ml balloon volume insufflation).

The absence of interaction between the groups (balloon inflation up to 4 ml or 7 ml) and the moment of the experiment suggest that the relationship between cytokines level and ICP elevation presents a plateau, or a ceiling effect. In other words, cytokines level varies in response to ICP elevation up to a certain level, above which no additional variation may occur.

The relationship between ICP and cytokines levels was statistically significant up to the moment in which the balloon was deflated and an ICP fall was observed. From there, the relationship was not significant. We speculate the variation of cytokine levels occurs along with the ICP due to an inflammation mechanism triggered by the primary lesion of the brain tissue or secondary to the ICH. After balloon deflation, there is a reduction in the ICP, but the inflammatory process secondary to brain injury remains, maintaining the variation of cytokine levels established in the first moments of the experiment.

The use of some of those cytokines as biomarkers has already been shown in previous studies. Stein et al. [14] used IL-8 and TNF-α to predict ICH and cerebral hypoperfusion after TBI with substantial specificity (81–95%).

The main limitation of neuroinflammation studies is the fact that authors assess the relationship between cytokines selected a priori and specific clinical variables such as intracranial hypertension and hypoxemia, or the prognostic value of these mediators. In these studies, univariate correlations between a given biomarker and a clinical outcome often lead to inferences regarding the biological action of the selected cytokines. This approach may be flawed because brain tissue injury is the common trigger for cytokine production, as appears to be the case in our study. More than studies of individual cytokine profiles associated with specific variables, there are suggestions in the literature that information from the biomarkers profile should come from an inflammatory profile pattern [15, 16]. In this perspective, a “cytokine loading score” was proposed in which persistent elevations of IL-1β, IL-6, IL-8, IL-10, and TNF-α throughout the first year post-TBI would be predictive of prognosis 6 and 12 months after the event [17, 18].

Our experiment isolated most of the confounding factors present in cytokine studies. The development of another experimental model changing the balloon positioning site to the extradural space to reduce brain tissue injury may complement the findings of the present study. Moreover, the addition of long-term timepoints could give important information regarding the prognostic significance of cytokines variation. Finally, our experiment did not include a sham surgery arm. However, since most interleukin have a short half-life of minutes, this should not have an important impact [19–22].

The lack of correlation in some variables might be explained by the limited sample size. This was an exploratory study without adjustment for multiple comparison, but all hypotheses were prespecified. Moreover, most of the results would remain significant even with statistical corrections for multiple comparisons. Continuous assessments would also provide a more detailed data for the trend that we found for each cytokine. The use of other variables, including the tissue oxygen pressure and cerebral perfusion pressure can also be used in future experiments to give more insights on the value of blood biomarker modifications.

## Conclusions

The serum concentration of cytokines varied in response to intracranial hypertension. The study demonstrated the specific changes in each cytokine after intracranial hypertension and provides key information to guide neuroinflammation clinical research. The proposed experiment was successful as an animal model to the study of neuroinflammation biomarkers, although some improvement may be needed to verify the existence of a dose–response relationship between ICP and cytokines variation.

## Supplementary Information

**Additional file 1.Supplement 1.** Sample characterization and baseline physiologic parameters.

## Data Availability

All data are available within the text.

## References

[1] Marcolini E, Stretz C, DeWitt KM (2019). Intracranial hemorrhage and intracranial hypertension. Emerg Med Clin North Am.

[2] Jha RM, Kochanek PM (2018). A precision medicine approach to cerebral edema and intracranial hypertension after severe traumatic brain injury: Quo Vadis?. Curr Neurol Neurosci Rep.

[3] Vincent JL, Bogossian E, Menozzi M (2020). The future of biomarkers. Crit Care Clin.

[4] Yan EB, Hellewell SC, Bellander BM (2011). Post-traumatic hypoxia exacerbates neurological deficit, neuroinflammation and cerebral metabolism in rats with diffuse traumatic brain injury. J Neuroinflammation.

[5] Woodcock T, Morganti-Kossmann MC (2013). The role of markers of inflammation in traumatic brain injury. Front Neurol.

[6] Brough D, Rothwell NJ, Allan SM (2015). Interleukin-1 as a pharmacological target in acute brain injury. Exp Physiol.

[7] Naraoka M, Fumoto T, Li Y (2019). The role of intracranial pressure and subarachnoid blood clots in early brain injury after experimental subarachnoid hemorrhage in rats. World Neurosurg.

[8] Lafrenaye AD, Krahe TE, Povlishock JT (2014). Moderately elevated intracranial pressure after diffuse traumatic brain injury is associated with exacerbated neuronal pathology and behavioral morbidity in the rat. J Cereb Blood Flow Metab.

[9] de Andrade AF, Soares MS, Patriota GC (2013). Experimental model of intracranial hypertension with continuous multiparametric monitoring in swine. Arq Neuropsiquiatr.

[10] du Sert NP, Hurst V, Ahluwalia A (2020). The arrive guidelines 2.0: updated guidelines for reporting animal research. PLoS Biol.

[11] Marehbian J, Muehlschlegel S, Edlow BL (2017). Medical management of the severe traumatic brain injury patient. Neurocrit Care.

[12] Davanzo JR, Sieg EP, Timmons SD (2017). Management of traumatic brain injury. Surg Clin North Am.

[13] Hergenroeder GW, Moore AN, McCoy JP (2010). Serum IL-6: a candidate biomarker for intracranial pressure elevation following isolated traumatic brain injury. J Neuroinflammation.

[14] Stein DM, Lindel AL, Murdock KR (2012). Use of serum biomarkers to predict secondary insults following severe traumatic brain injury. Shock.

[15] Mazzeo AT, Filippini C, Rosato R (2016). Multivariate projection method to investigate inflammation associated with secondary insults and outcome after human traumatic brain injury: a pilot study. J Neuroinflammation.

[16] Helmy A, Antoniades CA, Guilfoyle MR (2012). Principal component analysis of the cytokine and chemokine response to human traumatic brain injury. PLoS ONE.

[17] Nwachuku EL, Puccio AM, Adeboye A (2016). Time course of cerebrospinal fluid inflammatory biomarkers and relationship to 6-month neurologic outcome in adult severe traumatic brain injury. Clin Neurol Neurosurg.

[18] Ferreira LCB, Regner A, Miotto KDL (2014). Increased levels of interleukin-6, -8 and -10 are associated with fatal outcome following severe traumatic brain injury. Brain Inj.

[19] Jones AT, Ziltener HJ (1993). Enhancement of the biologic effects of interleukin-3 in vivo by anti-interleukin-3 antibodies. Blood.

[20] Donohue JH, Rosenberg SA (1983). The fate of interleukin-2 after in vivo administration. J Immunol.

[21] Conlon PJ, Tyler S, Grabstein KH, Morrissey P (1989). Interleukin-4 (B-cell stimulatory factor-1) augments the in vivo generation of cytotoxic cells in immunosuppressed animals. Biotechnol Ther.

[22] Martin CE, van Leeuwen EMM, Im SJ (2013). IL-7/anti-IL-7 mAb complexes augment cytokine potency in mice through association with IgG-Fc and by competition with IL-7R. Blood.

