# Supplementary material for: Inflammatory markers assessment in an animal model of intracranial hypertension: a randomized trial
Source: Intensive Care Med Exp. 2021 Aug 23;9:42. doi: 10.1186/s40635-021-00408-5 (PMC8380614; doi:10.1186/s40635-021-00408-5)
Supplement: Supplementary file 1 — Additional file 1.Supplement 1. Sample characterization and baseline physiologic parameters. [file 40635_2021_408_MOESM1_ESM.docx]

| *Supplement 1. Sample characterization and baseline physiologic parameters* | | | | | |
| --- | --- | --- | --- | --- | --- |
| **Variable** | **Group** | | **Total (20)** | **p-value** |  |
|  | **4mL (10)** | **7mL (10)** |  |  |  |
| **Female** | 7 (70,0) | 3 (30,0) | 10 (50,0) | 0,074 |  |
| **Weight (Kg)** | 24,1 ± 8,9 | 21,6 ± 7,4 | 22,9 ± 8,1 | 0,504 |  |
| **Physiology** |  |  |  |  |  |
| SBP (mmHg) | 104,2 ± 19,7 | 108,5 ± 12,7 | 106,4 ± 16,3 | 0,570 |  |
| DBP (mmHg) | 61,2 ± 12,8 | 72,6 ± 11,4 | 66,9 ± 13,1 | 0,049 |  |
| MAP (mmHg) | 76,1 ± 15 | 85,7 ± 10,4 | 80,9 ± 13,5 | 0,111 |  |
| EtCO2 (mmHg) | 43,6 ± 5,2 | 40,5 ± 4,2 | 42,1 ± 4,9 | 0,162 |  |
| Temperature (ºC) | 37,9 ± 1,1 | 37,7 ± 1,1 | 37,8 ± 1,1 | 0,643 |  |
| Heart rate (bpm) | 97,2 ± 32,1 | 107 ± 24,4 | 102,1 ± 28,2 | 0,452 |  |
| SatO2 (%) | 97,8 ± 1,2 | 97,5 ± 1 | 97,7 ± 1,1 | 0,552 |  |
| Brain temp. (ºC) | 37,5 ± 1,1 | 37,8 ± 0,8 | 37,7 ± 0,9 | 0,484 |  |
| PtiO2 (mmHg) | 5,1 (2,0 – 38,5) | 6,3 (1,0 – 12,8) | 6,3 (1,4 – 17,7) | 0,327 |  |
| Data were presented as mean ± standard deviation, except for gender – n(%) – and PtiPO2 – median (quartiles). SPB: systolic blood pressure; DBP: diastolic blood pressure; MAP: mean arterial pressure; EtCO2: End-tidal CO2; ⁠ PtiO2⁠: tissue oxygen pressure | | | | | |
